# Restoration of Abnormal Gum Tissue

**Published:** 1918-09

**Authors:** A. C. Fones


### RESTORATION OF ABNORMAL GUM TISSUE

BY A. C. FONES, D.D.S.

The five essentials of cell life are air, water, food, a proper temperature and the elimination of waste products. Deprive the cells of a tissue of one of these essentials and we inhibit their power to properly functionate. Metabolism also is interfered with, both anabolism and catabolism. With metabolism interference other essentials are not fully utilized and if the condition prevails for a sufficient length of time the resistance and vitality of the cells are lowered and, if in proximity to bacteria, infection may become a simple matter.

Packed around the necks of the teeth in unsanitary mouths is found food *débris* and millions of bacteria. In the subgingival space where the tissues are protected by a thin layer of nucleated cells only, the constant pressure of the toxins of bacteria and the end products of decomposing food cause an irritation to the delicate tissue. The irritation is extended to the papillae which are very close to the surface. The capillaries in the papillae become enlarged by the engorgement of blood, which being unable to flow readily now moves slowly in the peripheral circulation. With the increase of blood and the sluggish flow comes an increase of temperature. Waste products are not being readily carried away from the lymph spaces and the cells are partially suffocated in their own excreta. The blood in the capillaries becomes surcharged with carbon dioxide and the gums take on a dark-red color. A condition of suboxidation now exists, metabolism is interfered with, the power to fight and resist is lost in this depressing environment and the invasion of the bacteria is now but a comparatively simple matter.

Where we find mouths with the gum tissue of a dark-red color, shiny in appearance and which looks as if it would bleed almost on touch, the corneous layer of the mucous membrane is apt to be very thin and easily penetrated by bacteria. To re-establish health in this tissue is not a difficult matter unless a diseased condition of a serious nature is developed. First, establish cleanliness on all surfaces of all the teeth, especially the necks of the teeth. This removes the exciting cause. Next the gums should be stimulated by the use of the tooth brush, carefully at first, so as not to wound the mucous membrane, but to encourage a flow of blood from the congested capillaries under the borders of the gingivae. This blood letting and surface stimulation will relieve the congestion, the capillaries will soon assume their normal size, the waste products will be rapidly carried away, a proper temperature established and oxygen, water and food will be in demand.

Under a daily stimulation of the gum tissue by use of the tooth brush, the corneous layer of the mucous membrane is thickened and forms, as it were, an armor plate for the underlying tissues. The blood flow is quickened and the lymphatics readily drain the lymph spaces. The stimulation is felt in the pericementum by establishing a free flow of blood. The question has arisen in my mind whether there is not a change in the subgingival tissue under this gum stimulation.

The ordinary gums may be dried, but will be found to weep at their borders in a very short time, for serum is being forced out through the walls of the tissue lining the subgingival space. In a mouth with well-brushed gums the gingival border will remain dry for a long period and, although the subgingival space may be moist from mucous, the weeping has stopped. Have these nucleated cells built a thin corneous layer over themselves under the gum stimulation, or has the pressure from within the lymph spaces been decreased so that it no longer forces its surplus material through the walls of the subgingival space? At any rate, we may feel assured that under prophylaxis and gum brushing the soft tissues surrounding the teeth may be kept in a state of health indefinitely and the dangers of bacterial invasion at this point greatly lessened.—Extract from August *Dental Items of Interest.*

